# Exposure notification system activity as a leading indicator for SARS-COV-2 caseload forecasting

**DOI:** 10.1371/journal.pone.0287368

**Published:** 2023-08-18

**Authors:** Eliah Aronoff-Spencer, Sepideh Mazrouee, Rishi Graham, Mark S. Handcock, Kevin Nguyen, Camille Nebeker, Mohsen Malekinejad, Christopher A. Longhurst

**Affiliations:** 1 School of Medicine, Division of Infectious Diseases and Global Public Health, University of California San Diego, La Jolla, CA, United States of America; 2 University of California Los Angeles, Los Angeles, CA, United States of America; 3 Herbert Wertheim School of Public Health and Longevity Sciences, University of California San Diego, La Jolla, CA, United States of America; 4 University of California San Diego Health, San Diego, CA, United States of America; 5 California Department of Public Health, Sacramento, CA, United States of America; 6 Department of Epidemiology and Biostatistics, University of California, San Francisco, San Francisco, CA, United States of America; China University of Mining and Technology, CHINA

## Abstract

**Purpose:**

Digital methods to augment traditional contact tracing approaches were developed and deployed globally during the COVID-19 pandemic. These “Exposure Notification (EN)” systems present new opportunities to support public health interventions. To date, there have been attempts to model the impact of such systems, yet no reports have explored the value of real-time system data for predictive epidemiological modeling.

**Methods:**

We investigated the potential to short-term forecast COVID-19 caseloads using data from California’s implementation of the Google Apple Exposure Notification (GAEN) platform, branded as CA Notify. CA Notify is a digital public health intervention leveraging resident’s smartphones for anonymous EN. We extended a published statistical model that uses prior case counts to investigate the possibility of predicting short-term future case counts and then added EN activity to test for improved forecast performance. Additional predictive value was assessed by comparing the pandemic forecasting models with and without EN activity to the actual reported caseloads from 1–7 days in the future.

**Results:**

Observation of time series presents noticeable evidence for temporal association of system activity and caseloads. Incorporating earlier ENs in our model improved prediction of the caseload counts. Using Bayesian inference, we found nonzero influence of EN terms with probability one. Furthermore, we found a reduction in both the mean absolute percentage error and the mean squared prediction error, the latter of at least 5% and up to 32% when using ENs over the model without.

**Conclusions:**

This preliminary investigation suggests smartphone based ENs can significantly improve the accuracy of short-term forecasting. These predictive models can be readily deployed as local early warning systems to triage resources and interventions.

## 1. Introduction

In the 1530 rhymed account of epidemic disease, Giralamo Fracastoro gave name to the great pox “Syphilis sive morbus Gallicus” (Syphilis or the French Disease) and perhaps the earliest Western account of what is now referred to as *contact tracing (CT)* [1]. Even as many nations have implemented manual CT programs in response to COVID-19, SARS-COV-2 dynamics and global reach have quickly overwhelmed most traditional approaches, though there are significant bright-spots [2]. To meet this challenge, new solutions that synergize with CT have been developed employing surrogates of contact such as anonymous proximity notification utilizing smartphones; creating digital systems that emulate and potentially go beyond traditional public health practices [3]. These systems have now been deployed globally, yet there remain significant gaps in our understanding of impact. Further, there are no reports demonstrating that privacy preserving strategies that are foundational to the GAEN platform may still allow for epidemiological forecasting or spatiotemporal prediction models.

In April 2020, Google and Apple jointly released the Google Apple Exposure Notification (GAEN) API built with the “Private Automated Contact Tracing” (PACT) protocols [4], to scale contact tracing through smartphone-based proximity sensing and ENS [5]. Early evidence supported epidemiological impact [6], leading to reports that modeled EN’s effect in the context of other public health interventions [7, 8]. California leveraged the GAEN platform for its exposure notification system, and launched CA Notify statewide on Dec 10, 2020 [9]. As of this writing, the system has been activated nearly 17million times. With over one year of data, we can now explore the potential contribution of EN data for predictive modeling.

### 1.2 Evidence before the study

There have been significant advances in how non-exposure notification data are used for epidemiological forecasting, however, to date there are no reports employing EN data to predict future caseload counts. Numerous other forecasting models have been deployed that can be categorized into three different model types: i) Susceptible-Exposed-Infected-Recovered (SEIR) or Susceptible-Infected-Recovered (SIR) models [10, 11]; b) Agent-based models [12]; and c) Curve-fitting models [13–15]. The majority of the predictive models use parameters such as number of confirmed cases and deaths, masking guidelines, vaccinations and other public health intervention measures for prediction [16]. However, these types of data are commonly incomplete, hard to collect, and error-prone, forcing models to make a variety of assumptions to improve accuracy of the predictive models and to avoid overfitting. Moreover, other factors such as i) hospital settings/capacity; ii) test capacity/rate (on a daily basis); iii) demographics; iv) population density; v) income/poverty are still unexploited in most of the COVID-19 prediction models [17]. At the same time, data collected from ENs such as CA Notify, while limited by privacy preserving policy, still have the potential to provide massive amounts of data that are available in near-real time, because they are collected faster and updated more frequently than traditional sources.

### 1.3 Added value of this study

This study presents a first examination of how EN data may be employed to predict future case rates. Our analyses go beyond existing modeling approaches, which have only considered ENs impact estimation, and we show that such data are highly predictive of near-term cases. Future work should employ more detailed modeling methods, add other time series to enhance prediction power, and explore longer prediction horizons.

### 1.4 Implications of all the available evidence

New digital public health tools such as EN systems present new opportunities to improve health outcomes and disease prediction, yet the limitations and potential hazards of data use remain under explored.

## 2. Methods

In this study, our goal was to investigate the potential to short-term forecast COVID-19 caseloads using data from the implementation of CA Notify in California. Aggregate system statistics (2020–2021) were extracted and analyzed for potential predictive power of EN activities to future caseload counts.

Anonymous aggregate state level code usage data (described below), and state and county level exposure notification web traffic statistics were obtained under the data use agreement with the California Department of Public Health (CDPH). This study was deemed to be IRB exempt (no human subjects research from institutional review by the Committee for the Protection of Human Subjects at the University of California San Diego. The data were collected and shared in compliance with the CA Notify privacy policy. Reported case counts (containing only test report date) and hospitalization data were obtained from open access CDPH data [18]. We plotted raw data and applied a 7-day right-aligned moving average, due to known weekly reporting artifacts and potential intrinsic positive time correlations between case counts, which initialize system activity.

The collection of case count data fall under mandatory reporting guidelines for infectious diseases, however there are a number of days for which there are no reports for one or more days, with the subsequent report covering the case counts from the missing days. These “interval-reported” case counts were modeled via a non-homogeneous Negative Binomial regression model for the full data record with non-parametric trend and day-of-the-week effect. We stochastically imputed the individual non-reporting days from the model, conditioning that the totals by each "catch-up day" match the raw data.

To investigate the relationship of EN system activity and future case rates, we assume the following: 1- A user who has activated CA Notify and tests positive (index case) receives a code to enter into the system. 2- Code use triggers ENs to other users who have been in proximity for a certain time, and whose phones have exchanged anonymous keys. 3- Those who receive an EN are prompted to visit a non-searchable “hidden” CDPH website to access instructions on the next steps after potential exposure. Only daily total codes used (state aggregate) and count of visits to the notification webpage are captured. To model the relationship between **C** (Cases) and **E** (Exposure Notifications with visits to hidden website), we implemented a Bayesian predictive model for future values of **C** and showed that prediction could be improved by incorporating **E**.

We first developed a model for short-term forecasting of COVID-19 case counts based on work published by Oiviera et al [19]. We used a log-normal variant of their negative binomial model as the latter is over dispersed for our situation. We introduced a fixed-effect regressor on day-of-the-week into the mean function to account for known reporting artifacts. This model was used to forecast case counts 1–7 days in advance of the reporting data. Next, we modeled exposure notifications via a parallel log-normal autoregressive process with its own fixed-effect day-of-the-week regressor for reporting artifacts. The mean of log **C** on day *t* was then regressed on the underlying exposure process on day *t* as well as the prior 6 days, with corresponding coefficients ${\left\{v_{l}\right\}}_{l=0}^{6}$. The posterior distributions of those coefficients yield information on the forecasting influence of **E**. Values of **$V_{l_{\{}}$** greater than zero with high posterior probability provide prima facie evidence of the forecasting value of **E** *l* days in the past. We fit the model using Bayesian inference, implemented via the Markov Chain Monte Carlo method.

To compare the forecasting performance of the models with and without **E,** we consider forecasting days based on their history. Specifically, we fit the model based on data from day 1 to day *t* and then forecast days *t* + 1, *t* + 2, … *t* + 7. Each of these forecasts can then be compared to the recorded case counts for those days. To quantify performance, we computed the mean-squared error (MSE) and the mean absolute percentage error (MAPE) of the posterior mean forecast from that recorded over the days *t* = 150, 151, …. ,388.

The methods were implemented in R and Stan and we have made the code available [20].

## 3. Results

**Fig 1:**
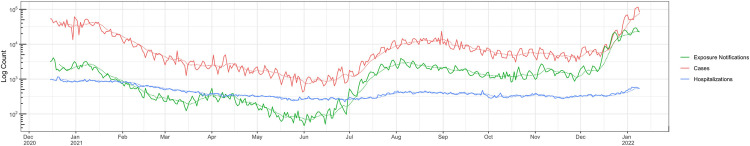
Raw and 7-day averaged data plotted versus time. Note initial equilibration time from Dec 10 launch into January, CA Notify proximity threshold setting change on March 17, 2021 date that led to a brief artificial increase in EN activity. Likewise, a server outage April 1, 2021 and October 11 to 19, 2021 led to a number of days without code issuance.

Fig 1 shows the raw and 7-day average for EN, reported case counts and hospital admissions from Dec 15, 2020 to Jan 9, 2022. Noting artifacts, early system equilibration in Dec 2020- Jan 2021 and change in CA Notify risk model settings in March (i.e., resulting an increased number of ENs being sent out per code used), we observe an intuitive temporal relationship whereby increases in EN activity are seen to precede reported case increases, and more subtly hospitalizations; notification inflection is observed prior to case peaks, and the downward trend of activity presages declining case rates.

**Fig 2:**
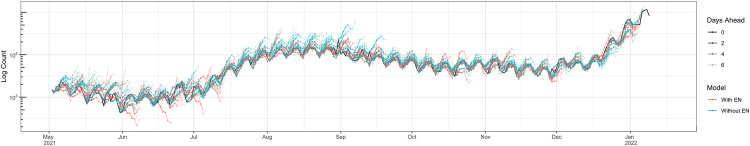
Raw cases and forecasts (1 to 7 days ahead) based on a model with and without exposure notifications.

Fig 2 depicts a sequence of forecasts made using each model. The black line shows **C** from late March 2021 to early January 2022. For each sequential day in this range, both models were fit using all data up to that day, then forecasts were made for the subsequent 7 days and compared against the actual values of **C**. The mean squared prediction error of the base model (without **E**) ranged from 0.05 at 1 day ahead to 0.38 at 7 days ahead, while that of the model with **E** ranged from 0.04 to 0.26. The mean absolute percentage error of the base model ranged from 1.0% to 6.4% and that of the model with E ranged from 1.8% to 5.0%. The figure shows each group of forecasts connected back to the day on which the forecast is made. Note that while a few of the predictive paths from the model with **E** strongly diverge, the majority of them appear to adhere more closely to **C** than the forecasts from the model without **E**.

**Fig 3:**
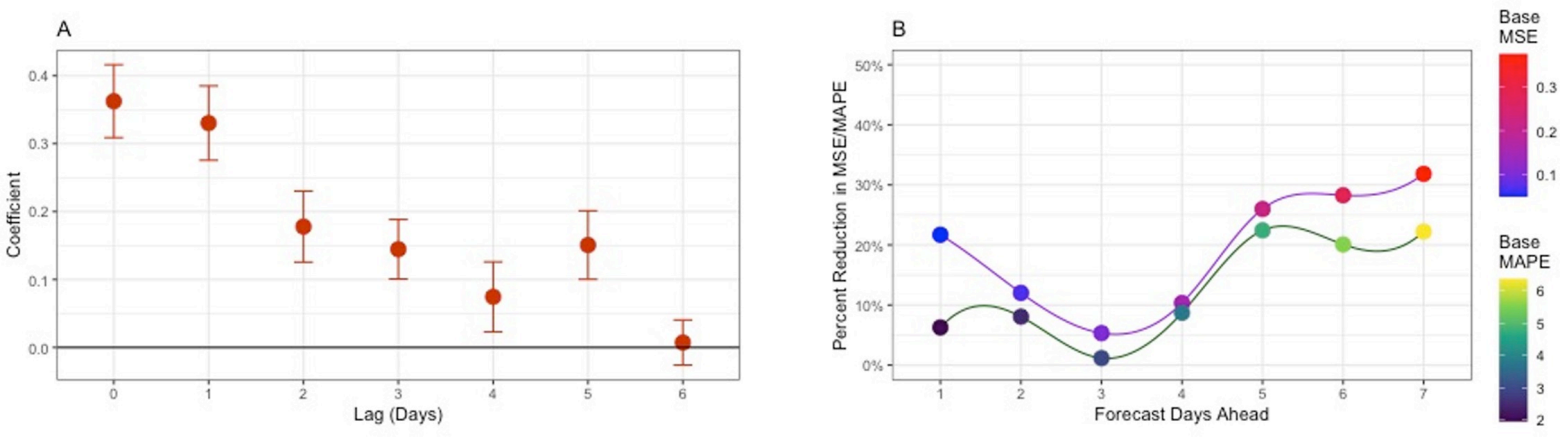
(**A**) Credibility intervals for the lagged exposure notification coefficients (Median +/- SD). Positive posterior values for lags > 0 indicate a positive association between case counts and previous ENs. (**B**) Percent reduction in MSE and MAPE of forecast by number of days ahead. The model using ENs performed significantly better than without.

To characterize this observable trend, we estimated *v*_*l*_ for 0–6 days of lag (Fig 3A presents the credibility intervals). The estimated posterior probability that at least one of the coefficients corresponding to positive lags was greater than zero was one. Fig 3B plots the ratio of the forecast MSE and MAPE for the model with **E** to those without for 1 to 7 days delay. We see that the incorporation of **E** substantially improves forecast accuracy. Specifically, the MSE is reduced by a minimum of 5% at 3 days ahead up to a maximum of 32% at 7 days ahead, while the MAPE is reduced by a minimum of 1.2% at 3 days ahead up to a maximum of 22.4% at 5 days ahead.

## 4. Discussion and a conclusion

We report the first analysis of an EN-based prediction model to short term forecast COVID-19 reported cases in California. Our approach extended an existing method that utilizes only prior case counts to predict future case counts, to usage of ENs for the prediction. With this method, we measured the change in forecasting accuracy 1–7 days in the future. We found that the addition of EN data to the previously validated case-only model improved mean squared prediction error by a modest 5% at 3 days up to 32% at 7 days, the longest horizon explored in this work. The mean absolute percentage error of prediction ranged from 1.2% to 22%. This indicates that near past EN is related to near future case counts and that its inclusion can improve the forecasting accuracy of case counts 1 to 6 or more days into the future.

In this study we compared omnibus measures of the performance of a model including EN data to a case-only model. This considers average performance over time. An complementary comparison could examine the performance of the models during sudden changes in the pandemic dynamic (e.g. in Fall 2020, when cases exploded or in December 2021 with the rise of omicron). Focusing our attention on the times of change in dynamics, sometimes visible in Figs 2 and S1 depicts model function in the period just before and during the rise of omicron (December 2021 to early 2022). For clarity, it shows only the daily recorded cases and 7-day predictions with and without EN (that is, forecasts only using information available 7 days prior). Here, we see a period of stability before 12/18 where both models accurately capture the stable dynamics. The next period, we now know, saw the rapid omicron rise. Forecasts using EN information match this rise, mimicking the recorded cases (S1 Fig, green versus black), whereas the model without EN struggles to predict future rates, and is consistently below until the New Year (S1 Fig, blue versus black). Note that this shows that our modeling of EN is effective, not just that EN is a leading indicator of cases (See the far right of Fig 1). The comparison to the non-EN forecast is important as it shows that a case-only model underperforms when EN is not used.

While it is possible to compare other periods of stable dynamics and changing dynamics, and to optimize the model fit to better forecast during periods of changing dynamics, the endogeneity of the phase (stable verses changing) makes this challenging and left for future work.

Other predictive models of COVID-19 caseload have been published during the pandemic [21], and there have been numerous attempts to model the impact of ENs, yet none have reported incorporating EN data for caseload prediction [6, 7, 22]. Thus, we add a new dimension to this toolkit by introducing EN as a predictor of the case number in the near term by extending a case forecasting model to include EN and reporting artifacts.

This study has limitations. First, case counts are reported based on test report rather than collection date. Second, there are subsequent system delays in code generation and use, which impact the time to ENS generation. Finally, this study purposely used a simple forecasting model relying only on case counts and ENs as a first and clear step to assess the viability of incorporating ENS data into predictive methods. Future work should examine more advanced models to handle higher dimensional data to incorporate more predictors (e.g., daily test rate, mobility, vaccinations) as well as more diverse datasets from different geographic locations which had different populations, intervention policies, for example.

### 4.1 Conclusion

In conclusion, this study presents preliminary evidence that smartphone-based EN can predict case count changes in the near term, a function that is beyond its primary public health role as a digital exposure notification system to augment traditional contact tracing. Such predictive models can be readily deployed as local early warning systems and integrated with other signals to triage resources and interventions. Future directions should expand simulations to incorporate more features such as mobility, daily tests, vaccination, and other key predictors while exploring longer prediction horizons.

### 4.2 Data sharing

Anonymous, aggregate data without specific geographic information were obtained by agreement with the California Department of Public Health (CDPH), published under Public Health Exemption, and in compliance with the CA Notify privacy policy. The data were analyzed in accordance with a Data Usage Agreement between the CDPH and UCSD. The raw data used in this study are not currently released to the general public in accordance with applicable policy and regulations. Data requests may be submitted to the California Department of Public Health.

## Supporting information

S1 Fig Recorded cases versus EN 7-day forecasts. Recorded (black) versus the seven day predicted cases with EN (green) and without EN (blue) for 12/11/2021-1/8/2022.
(TIFF)
