# Supplementary figures and images for: Exposure notification system activity as a leading indicator for SARS-COV-2 caseload forecasting
Source: PLoS One. 2023 Aug 18;18(8):e0287368. doi: 10.1371/journal.pone.0287368 (PMC10437830; doi:10.1371/journal.pone.0287368)

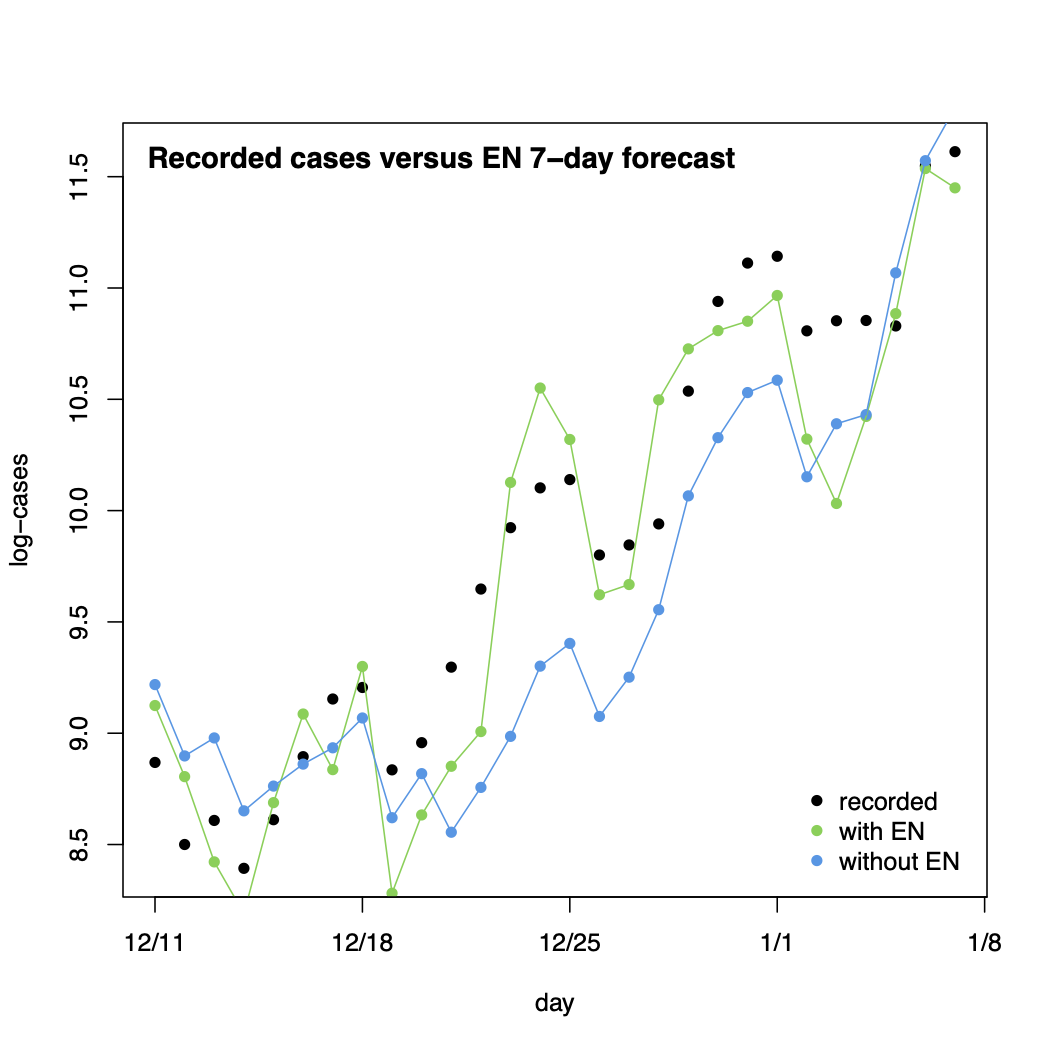

Supplement: S1 Fig — Recorded (black) versus the seven day predicted cases with EN (green) and without EN (blue) for 12/11/2021-1/8/2022. (TIFF) [file pone.0287368.s001.tiff]
